# Treatment outcomes of 156 patients with cervical esophageal cancers treated with definitive radiation therapy- A single-institution experience of a rare cancer

**DOI:** 10.3389/fonc.2022.929583

**Published:** 2022-08-18

**Authors:** Xiaofei Zhang, Xumeng Fang, Peiyi Liu, Di Liu, Huanjun Yang, Weixin Zhao, Fengtao Su, Kuaile Zhao

**Affiliations:** ^1^ Department of Radiation Oncology, Fudan University Shanghai Cancer Center, Shanghai, China; ^2^ Department of Oncology, Shanghai Medical College, Fudan University, Shanghai, China; ^3^ Department of Orthopedics, TongRen Hospital, School of Medicine, Shanghai Jiao Tong University, Shanghai, China; ^4^ Cancer Institute, Fudan University Shanghai Cancer Center, Shanghai Medical College of Fudan University, Shanghai, China

**Keywords:** cervical esophageal carcinoma, three-dimensional radiotherapy, radical radiotherapy, involved field irradiation, failure pattern

## Abstract

**Purpose:**

Esophageal cancer is the most prevalent malignant tumor. The incidence of cervical esophageal cancer is low and there are insufficient data on the efficacy of radical radiotherapy. The purpose of this study was to clarify the efficacy with radical IFI radiotherapy, to analyze the pattern of initial lymph node metastasis and recurrence under the new lymph node zoning of esophageal cancer.

**Methods:**

We reviewed cervical esophageal cancer treated with radical radiotherapy. The inclusion criteria were diagnosis of esophageal cancer by pathology; receiving radical radiotherapy or chemoradiotherapy; tumor location in accordance with definition of cervical esophageal cancer. Three dimensional radiotherapy was used. The target area was IFI.

**Results:**

156 patients entered the final analysis. The proportion of no failure was 42.31%, local esophageal failure was 30.13%, in-field lymph node metastasis was 10.26%, out-field lymph node metastasis was 1.28% and distant organ metastasis was 23.72%, second primary tumor was 2.56%. The median OS and DFS was 49.0 months (35.27-62.73) and 31.0 months (14.47-47.53). The results of initial LN metastasis pattern analysis showed the supraclavicular and upper mediastinum were the main sites of cervical esophageal cancer metastasis. In patients with recurrent LN, the results showed that the cervical, supraclavicle, upper mediastinum and abdomen were the main sites of recurrence.

**Conclusion:**

Our study is a retrospective study of a large sample of radical radiotherapy for cervical esophageal cancer. Failure in irradiation field is the main failure pattern. Concurrent radiotherapy and chemotherapy under IFI radiation is a considerable treatment option for cervical esophageal cancer.

## Background and purpose

Esophageal cancer is one of the most prevalent malignant tumors in China and worldwide (1, 2). The incidence of cervical esophageal cancer is low compared to that of lower esophageal cancer (3), accounting for approximately 5% of all esophageal cancers. Therapeutically, cervical esophageal cancer often lacks adequate margins and undergoes laryngopharyngectomy with a high rate of postoperative complications; therefore, radical radiotherapy is the standard treatment for cervical esophageal cancer (4, 5). Due to the low incidence of cervical esophageal cancer, there are insufficient data on the efficacy of radical radiotherapy for cervical esophageal cancer.

### Efficacy of radiotherapy for cervical esophageal cancer

There are inconsistent results on the prognosis of radiotherapy for cervical esophageal cancer (6–11). Some studies have suggested that the lymphatic drainage of the cervical esophagus is rich and prone to invade surrounding anatomical structures (e.g., cricoid and thyroid cartilage) and metastasis, making the prognosis after radiotherapy worse than that of other parts of the esophagus (6). However, some studies have concluded that the biological behavior of the cervical esophagus differs from that of the lower esophagus or the gastroesophageal junction, being locally invasive but less prone to distant metastases, and that its treatment protocol should refer to head and neck cancer tumors. In addition the sample size of previous published studies is limited and patients with both thoracic and cervical were included (6, 7), therefore data on the efficacy of radical radiotherapy for cervical esophageal cancer are not sufficient.

### Target areas of radiotherapy for cervical esophageal cancer

Radical radiotherapy is the standard treatment for cervical esophageal cancer (4, 5). However, whether prophylactic lymph node irradiation (ENI) should be performed has been controversial, especially for the cervical and upper thoracic esophageal cancer. Our previous study reported the outcome of 53 patients with various segments of esophageal cancer treated with radical radiotherapy with involved field irradiation (IFI). The results showed a median survival time (OS) of 30 months and local failure in the radiation area in 85% of patients, suggesting that ENI is not necessary for overall esophageal cancer (12).Yamashita also found that ENI did not help to improve OS and resulted in higher treatment-related mortality (13). In one of our previous retrospective studies, 169 squamous carcinomas of the cervical and upper thoracic esophagus were irradiated with or without ENI. OS, in field recurrence rates and distant organ metastasis rates were similar in both groups (14). Thus, for cervical and upper thoracic esophageal cancer overall, ENI did not significantly improve OS in patients with cervical and upper thoracic esophageal cancer compared with IFI, but there are insufficient data with large sample on the efficacy of radical radiotherapy with IFI for cervical esophageal cancer only.

### Lymph node zoning in esophageal cancer

**Figure 1:**
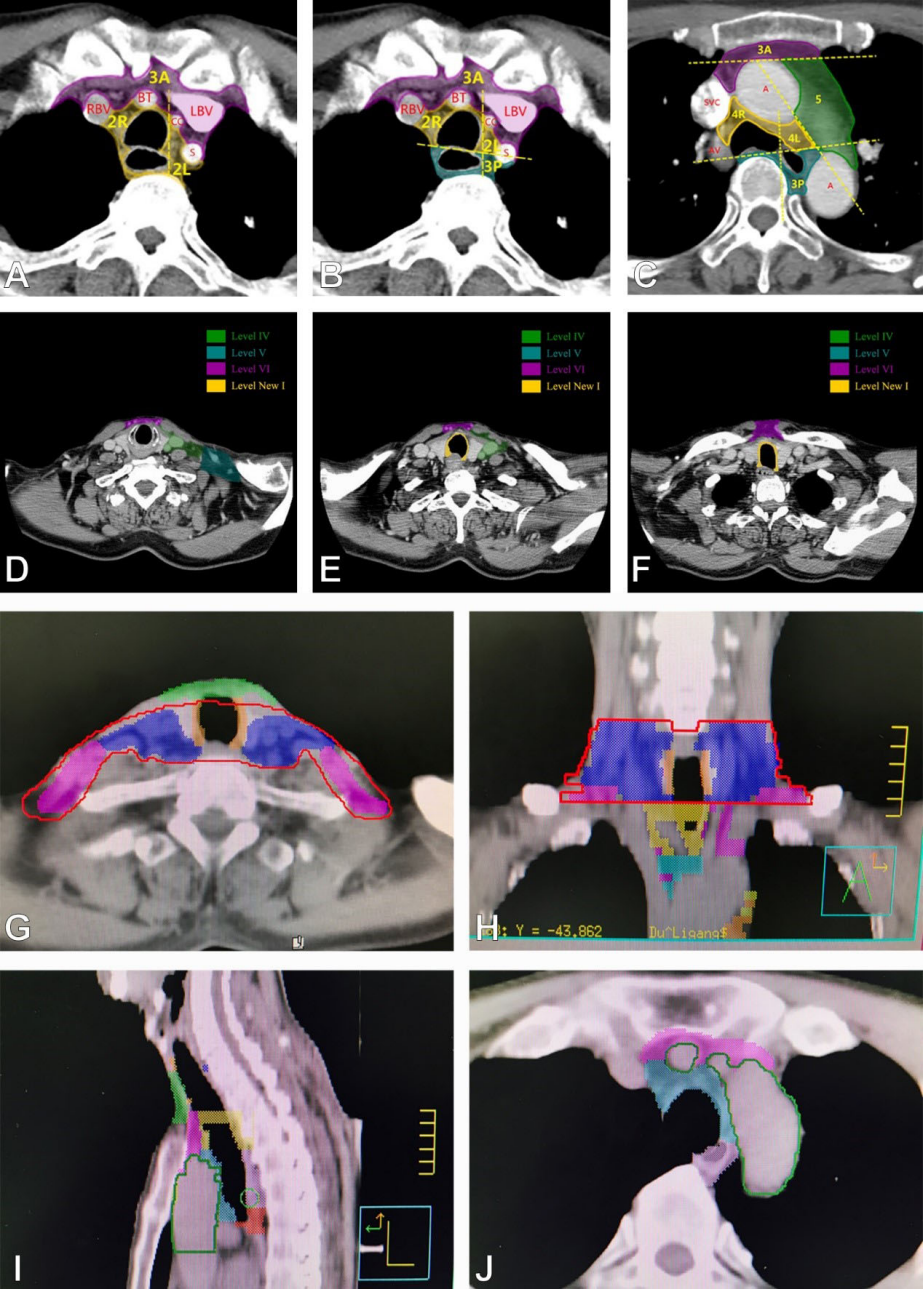
Zoning of lymph node regions. **(A–C)** show the new zoning of mediastinal lymph nodes of 3A,3P,2R,2L and 5. **(D-F)** show the new zoning of supraclavicular lymph nodes: IV, V, VI, new1. **(G, H)** show the entire new zoning of supraclavicular region in transverse and coronal positions. **(I)** shows the entire new zoning in sagittal positions. **(J)** shows the new zoning of 3A,4 and 3P.

In recent years, few atlas have been published on lymph node mapping in esophageal cancer. The guide of the Japanese Society for Esophageal Disease (JSED) is based on surgical anatomical structures such as blood vessels, muscles and even nerves, which are not easily visible in CT images and are more difficult to use for preoperative evaluation. In clinical practice, the CT-based outline of the cervical RTOG consensus guidelines (15) and the IASLC guidelines for thoracic lymph nodes (16) are widely used for esophageal cancer. However, they cannot be well distinguished in border region of the cervicothoracic. To solve this problem, our center published a new lymph node zoning method for esophageal cancer in a previous study (17), which integrated the RTOG consensus guidelines for cervical lymph node level delineation (15) and the IASLC guidelines for thoracic lymph node level definition (16), and clearly described the border zone (supraclavicular region). We redefined zone VI in the RTOG consensus guidelines, which previously included cervical paraesophageal lymph nodes and anterior thyroid lymph nodes, and we separated the two parts by designating the anterior thyroid area as the new zone VI and the cervical paraesophageal lymph nodes as the new zone 1, so that the supraclavicular area was divided into four zones (IV, V, VI, and new 1). We redefined the upper boundary of zone 3P of the thoracic lymph nodes of the IASLC guidelines as the upper border of the aortic arch. Thus, zone 2 is posteriorly bounded by the paravertebral muscle and includes the paraesophageal lymph nodes. The other cervical lymph node levels are the same as the RTOG consensus guidelines, see **Figure 1**. the new esophageal cancer zoning atlas provides consistent information for the evaluation and multidisciplinary treatment of esophageal cancer in the non-surgical setting.

### Purpose of the study

The purpose of this study was to review the treatment information of cervical esophageal cancer treated with radical IFI radiotherapy at Fudan University Shanghai Cancer Center from 2007 to 2014, and to clarify the efficacy of cervical esophageal cancer, including overall survival (OS), progression-free survival (PFS), local recurrence-free survival (LRFS), and metastasis-free survival (MFS). Also to evaluate the recurrence pattern of cervical esophageal cancer under the IFI radiotherapy target area and to analyze the pattern of initial lymph node metastasis and recurrence of cervical esophageal cancer under the new lymph node zoning of esophageal cancer, so as to provide a large sample of treatment basis for radical radiotherapy of cervical esophageal cancer.

## Materials and methods

### Patient inclusion and study design

In this study, we reviewed cervical esophageal cancers treated with radical radiotherapy at the Fudan University Shanghai Cancer Center between 2007 and 2014. Cervical esophageal cancer was defined as tumor located between the cricopharyngeal muscle and the entrance to the thoracic cavity or an esophageal lesion 15-20 cm from the incisor in endoscopy. The inclusion criteria for this study were (1) definite diagnosis of esophageal cancer including esophageal intraepithelial neoplasia with canceration by pathological histology or cytology; (2) receiving radical radiotherapy or chemoradiotherapy; (3) tumor location in accordance with the above definition of cervical esophageal cancer; and (4) having clinical history information required for follow-up, including medical history, physical examination, laboratory examination, endoscopy, esophagography and chest computed tomography (CT) scan. Exclusion criteria were: (1) history of esophageal surgery prior to radiotherapy; (2) any previous anti-tumor treatment; (3) history of other malignant tumors in combination including tumor history of more than 5 years.

Patients were staged using the American Joint Commission (AJCC) 7th edition criteria. The initial staging was based on clinical history, physical examination, laboratory examination, endoscopy, esophagogram and CT, most patients have PET/CT for evaluation, and ultrasonic gastroscopy is not necessary. Informed consent was obtained from each patient. The study was conducted according to the guidelines of the World Medical Association and the Declaration of Helsinki, and the protocol was approved by the Ethics Committee of the Fudan University Shanghai Cancer Center (1203108-4).

### Treatment

Patient immobilization, simulation and treatment planning are performed using 3D radiation therapy techniques. The radiologists performed target delineation based on endoscopic, ultrasound, esophagogram and CT scan image data. All patients received 3D conformal or intensity-modulated radical radiotherapy with linear accelerator 6mv-x-ray irradiation, and the target area of radiotherapy was treated with IFI (12), which were outlined as follows: the tumor target area (GTV) included the visible foci of the tumor (primary esophageal foci + metastatic lymph nodes), the clinical target area (CTV) was to put GTV up and down for 3cm without external radiation all around, and the planned target area (PTV) was 1 cm outside the CTV (1.5 cm outside the cardia if the target area is located in the cardia).

### Follow-up information

After treatment, follow-up visits were performed every 3 months for the first 2 years, every 6 months for years 3 to 5, and annually thereafter. Follow-up visits include the patient’s emerging complaints, appropriate physical examination (e.g., palpation of supraclavicular lymph nodes). Other diagnostic tests as clinically indicated include CT scan of the chest, ultrasound from the supraclavicular region to the abdomen, esophagogram, and esophagography once a year.

### Outcome measures

In our analysis, treatment failure patterns were categorized as no failure, failure of the esophagus, failure of lymph nodes in field, failure of lymph nodes out field, distant organ metastasis and second primary tumor. For the failure pattern analysis of all lymph nodes, the lymph node regions were divided as shown in **Figure 1**. It should be noted that we optimized the supraclavicular region into four regions (IV, V, VI, and New 1) (17). The failure pattern was recorded with reference to CT (including positional CT), positron emission computed tomography (PET/CT), and endoscopic records before and after treatment, as well as medical history. OS is the time from the end date of radiotherapy until death from any cause. PFS is the time from the end date of radiotherapy to the date of recurrence, metastasis, or death, whichever occurs first. Metastasis-free survival (MFS) is the time from the end date of radiotherapy to the date of recurrence or death from any cause in any primary tumor area and regional lymph nodes or distant organs outside the irradiated area. Local recurrence-free survival (LRFS) is defined as the time from the date of completion of radiotherapy to the date of recurrence of the esophageal lesion or lymph nodes within the irradiated area or death from any cause.

### Statistical analysis

The Statistical Package for Social Sciences (SPSS version 20.0, IBM Corporation, Chicago, IL, USA) was used to analyze the data. All measurement data will be statistically described using the mean ± standard deviation. All counting data were statistically described using frequency. The Chi-square test was used to analyze the count data. Survival indicators will be analyzed using Kaplan-Meier method and Log-rank test.

## Study results

### Patient characteristics and treatment methods

In this study, a total of 156 patients who were treated at Fudan University Shanghai Cancer Center between 2007 and 2014 were collected. The median age of the patients was 60 years (range 44-76 years). There were 156 (100.0%) of squamous carcinoma. The tumor stages was as follows: stage I (n=8, 5.1%), stage II (n=24, 15.4%), stage III (n=105, 67.3%), and stage IV (n=19, 12.2%, with supraclavicular lymph node metastasis). The main characteristics are shown in **Table 1**.

**Table 1:** General information of patients.

		Parameters	%
Age(year)	Median	60	
	Range	44-76	
Gender	Male	118	75.6
	Female	38	24.4
Pathology	Squamous carcinoma	156	100
	Adenocarcinoma	0	0
Stage AJCC 7^th^	Stage I	8	5.1
	Stage II	24	15.4
	Stage III	105	67.3
	Stage IV	19	12.2
Radiotherapy dose (Gy)	Median	61.2	
	Range	44-72	
Dose fractionation regimen	Later course accelerated hyperfractionation	9	5.8
	whole course accelerated hyperfractionation	5	3.2
	Clinical study dose (61. 2Gy/34f)	65	41.6
	Other dosing modalities	77	49.4
Chemotherapy	No	19	12.2
	TP	46	29.5
	TF	27	17.3
	PF	50	32.1
	S1	6	3.8
	Other	8	5.1

### Treatment-related analysis

Radical radiation therapy was completed in 152 (97.4%) patients. All radiation treatments were performed using three-dimensional techniques. The median radiotherapy dose was 61.2 Gy (range 44-72 Gy) with a single fractionated dose of 1.5-2.2 Gy. The specific radiotherapy dose fractionation was as follows: (1) Later course accelerated hyperfractionation(41.1 Gy/23f, qd followed by 27 Gy/18f, bid, n = 9 patients); (2) whole course accelerated hyperfractionation(60 Gy/40f, bid, n = 5); (3) clinical study dose (61. 2Gy/34f, qd, n = 65); (4) other conventional dose (50.4-72Gy, 1.8-2.2Gy, qd, 5 times a week, n = 77). The median BED of 156 patients was 60Gy(50-72gy). 137 (87.8%) patients received concurrent chemotherapy (**Table 1**). The number of chemotherapy cycles of 131 patients receiving intravenous chemotherapy are showing in **Supplementary Table S1**.

### Initial lymph node metastasis pattern

**Figure 2:**
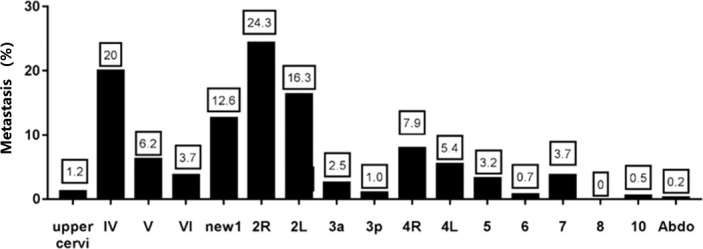
Metastasis rates by region in the initial lymph node metastasis pattern analysis.

Among the patients with initial lymph node metastasis, a total of 404 lymph nodes metastasized. The metastasis rates by region were 1.2% in the upper cervical, 20.0% in region IV, 6.2% in region V, 3.7% in region VI, 12.6% in region New 1, 24.3% in region 2R, 16.3% in region 2L, 2.5% in region 3a, 1.0% in region 3p, 7.9% in region 4R, 5.4% in region 4L, 3.2% in region 5, 0.7% in region 6, 3.7% in region 7, 0% in region 8, 10 0.5% and abdominal lymph nodes 0.2% (**Figure 2**). The results showed that the highest rate of lymph node metastasis was found in zones IV, V, 2 and 4, so supraclavicular and upper mediastinum were the main sites of esophageal cancer metastasis in the cervical esophageal cancer. In contrast, the lymph node metastasis rate was lower in the upper cervical, 3P, 6 and the abdominal.

### Outcome and prognosis

**Figure 3:**
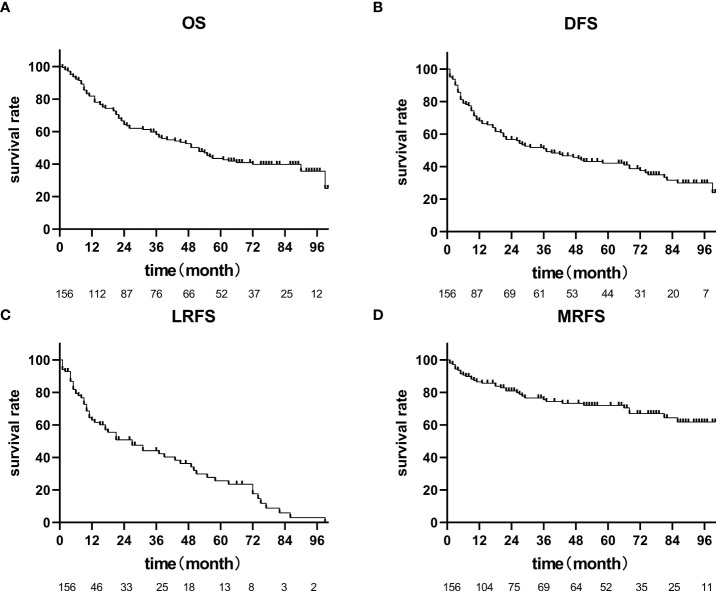
OS, PFS, LRFS, MRFS of 156 patients with cervical esophageal cancer treated with radical radiation therapy. **(A)** The median OS was 49.0 months (35.27-62.73), **(B)** Median DFS was 31.0 months (14.47-47.53), **(C)** The median LRFS was 21.0 months (8.22-33.77), **(D)** Median MFS was not achieved.

Until July 2020, there were 86 deaths and the median follow-up for the cohort was 35 months. The pattern of failure is shown in **Table 2**. The proportion of patients with no failure was 42.31%, the proportion of localized esophageal lesion failure was 30.13%, the proportion of lymph node metastasis in field was 10.26%, the proportion of out field lymph node metastasis was 1.28%, distant organ metastasis was 23.72%, and second primary tumor was 2.56%. The median OS was 49.0 months (35.27-62.73), with a 1-year OS rate of 70.51%, a 2-year OS rate of 55.13%, a 3-year OS rate of 48.08%, and a 5-year OS rate of 33.33%. Median DFS was 31.0 months (14.47-47.53), 1-year DFS rate was 55.13%, 2-year DFS rate was 43.59%, 3-year DFS rate was 38.46%, and 5-year DFS rate was 27.56%. The median LRFS was 21.0 months (8.22-33.77), with a 1-year LRFS rate of 48.08%, 2-year LRFS rate of 40.38%, 3-year LRFS rate of 32.69%, and 5-year LRFS rate of 17.31%. Median MFS was not obtained with a 1-year MFS rate of 92.30%, a 2-year MFS rate of 87.17%, a 3-year MFS rate of 76.92%, and a 5-year MFS rate of 61.54% (**Figure 3**). All AEs occurred during treatment are listed in **Tables 3**, **4**.

**Table 2:** First failure pattern.

First time failure pattern	N (%)*
No failure	66 (42.31)
Failure of the esophagus	47(30.13)
Failure of lymph nodes in field	16(10.26)
Failure of lymph nodes out field	2(1.28)
Distant organ metastasis	37(23.72)
Second primary tumor**	4 (2.56)

^*^Patients have various recurrences appearing overlapping, and the denominator is uniformly defined as 156 when we calculate the rate. **Second primary tumor does not include esophageal second primary tumor.

**Table 3:** Acute Treatment Toxicity.

Acute Treatment Toxicity	N (%)*				
	Grade 0	Grade 1	Grade 2	Grade 3	Grade 4
Hematological toxicity					
Leukopenia	28 (17.9)	47 (30.1)	49 (31.4)	27 (17.3)	5 (3.2)
Neutropenia	32 (20.5)	55 (35.3)	34 (21.8)	21 (13.5)	14(9.0)
Anemia	110 (70.5)	38 (24.6)	7 (4.5)	1 (0.6)	0 (0)
Thrombocytopenia	131 (84.0)	16 (10.3)	5 (3.2)	4 (2.6)	0 (0)
Nonhematological toxicity					
Fatigue	143 (91.67)	13 (8.3)	0 (0)	0 (0)	0 (0)
Nausea/vomiting	139 (89.1)	10 (6.4)	7 (4.5)	0 (0)	0 (0)
Esophagitis	47 (30.1)	57 (36.5)	49 (31.4)	3 (1.8)	0 (0)
Pneumonitis	101 (64.7)	25 (16.0)	27 (17.3)	3 (1.9)	0 (0)
Dermatitis	146 (93.6)	7 (4.5)	2 (1.3)	1 (0.6)	0 (0)
Hemorrhage	154 (98.7)	1 (0.6)	1 (0.6)	0 (0)	0 (0)
Other	154(98.7)	0 (0)	0 (0)	0 (0)	2 (1.3)+

Acute AE were defined as occurred during or within 6 months after radiotherapy. ^*^The denominator is 156 when we calculating the ratio. +Two grade IV adverse events were esophageal fistula.

**Table 4:** Chronic Treatment Toxicity.

Chronic Treatment Toxicity	N (%)*				
	Grade 0	Grade 1	Grade 2	Grade 3	Grade 4
Hematological toxicity					
Leukopenia	154 (98.7)	1 (0.6)	1 (0.6)	0 (0)	0 (0)
Neutropenia	154 (98.7)	1 (0.6)	1 (0.6)	0 (0)	0 (0)
Anemia	155 (99.3)	1 (0.6)	0 (0)	0 (0)	0 (0)
Thrombocytopenia	152 (97.4)	2 (1.3)	2 (1.3)	0 (0)	0 (0)
Nonhematological toxicity					
Fatigue	156 (100)	0 (0)	0(0)	0 (0)	0 (0)
Nausea/vomiting	156 (100)	0 (0)	0 (0)	0 0()	0 (0)
Esophagitis	148 (94.9)	6 (3.8)	1 (0.6)	1 (0.6)	0 (0)
Pneumonitis	134 (85.9)	15 (9.6)	5 (3.2)	2 (1.3)	0 (0)
Dermatitis	156 (100)	0 (0)	0 (0)	0 (0)	0 (0)

Chronic AE were defined as occurred after 6 months after radiotherapy. ^*^The denominator is 156 when we calculating the ratio.

### Pattern of lymph node recurrence

**Figure 4:**
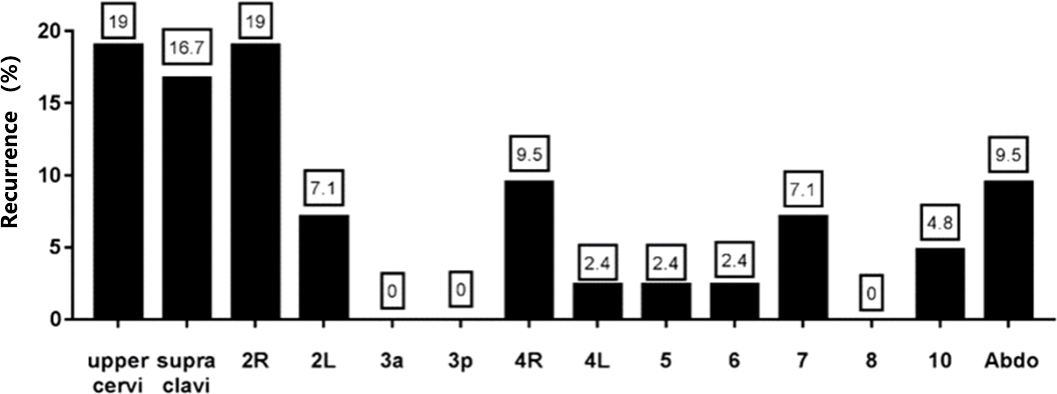
Lymph node metastasis pattern in patients with recurrent lymph nodes.

42 metastatic lymph nodes were identified in patients with recurrence. The distribution of recurrent lymph nodes was as follows, 19% in the upper cervical, 16.7% in the supraclavicular region, 19% in the 2R region, 7.1% in 2L, 0% in 3a, 0% in 3p, 9.5% in 4R, 2.4% in 4L, 2.4% in 5, 2.4% in 6, 7.1% in 7, 0% in 8, 4.8% in 10, and 9.5% in the abdominal lymph nodes (**Figure 4**). The results showed that the cervical, supraclavicular and upper mediastinum were the main sites of recurrence of cervical esophageal cancer, and the recurrence rate in the abdomen was also relatively high.

## Discussion

Previous studies have shown inconsistent prognosis in cervical esophageal cancer (6–10). Our results show that the prognosis of cervical esophageal cancer is acceptable, which we analyze has the following reasons, one being the position of cervical esophageal cancer is high. Radical dose radiotherapy can be given on the premise of ensuring the dose limit of endangering organs concerned in radiotherapy such as heart and lung, without large radiotherapy related side effects, so as to improve the curative effect. The second reason is the use of IFI in this study. We believe that for patients with cervical esophageal cancer, whose nutritional status is impaired due to long-term swallowing difficulties and whose bodies have limited to withstand intensive treatment, the use of radiation coverage with IFI may result in improved patient tolerance and thus improved completion rates of radiation therapy. The third reason is that all patients received 3D radiotherapy including intensity-modulated radiotherapy and conformal radiotherapy, and the proportion of patients receiving concurrent radiotherapy and the completion rate of radiotherapy was high. The fourth reason is that most patients (90.2%) received PET/CT, which allowed for more accurate staging and selection of subsequent treatment strategies. The fifth reason is that all patients were followed up regularly and on time according to the follow-up schedule mentioned in the methods section and the active treatment after subsequent relapse as in **Supplementary Table S2**.

In addition, our study showed that the lymph node metastasis pattern of cervical esophageal cancer and recurrence pattern was consistent with previous studies, and lymph node metastasis in the supraclavicular region and upper mediastinum remained the main areas of metastasis and recurrence of cervical esophageal cancer (18, 19). In addition, the most common failure pattern was in field failure, with 10.26% of lymph node recurrence in the esophageal lymph node field and 1.28% of lymph node recurrence out the field, so the irradiation pattern of IFI is sufficient and the necessity of ENI for cervical esophageal cancer is not significant.

For the high local failure rate, the reason may be the sensitivity of esophageal tumors to radiotherapy itself. The solution is to increase the radiotherapy dose and increase the radiotherapy sensitivity. For increasing the dose, previous studies have ended in failure (20–22). For increasing the dose, previous studies have ended in failure. The study of RTOG8501 showed that the 3-year OS, 5-year OS, local failure rate, and distant metastasis rates were better in the 50Gy radiotherapy group than in the 60Gy radiotherapy alone group, but the side effect rate was higher in the 50Gy radiotherapy group than in the 60Gy radiotherapy alone group (20). Based on the above results of RTOG8501 study, the efficacy of radiotherapy was not very satisfying, therefore, RTOG9405 study was proposed with increased dose. The radiation doses of the two groups were 50.4 Gy and 64.8 Gy respectively, and with same chemotherapy regimen of fluorouracil plus cisplatin. The results suggest that the 50.4 Gy dose group has better efficacy (21). Xu et al. Compared the efficacy of 60Gy radiotherapy dose and 50gy radiotherapy dose on esophageal squamous cell carcinoma with three-dimension radiation technology. Preliminary results showed no statistically significant differences between the two groups in terms of efficacy (22). Therefore, local dosing methods for functional images such as PET/CT are being discussed, and we are waiting for the results of these studies. In addition, the way to improve the local failure is to improve the radiotherapy sensitivity of esophageal cancer cells. Proton and other technologies may be better than photon. In addition, the combination of immunotherapy drugs, chemotherapy drugs and radiotherapy is also worth discussing. We are waiting for the results of these studies.

There are several limitations of our study. First, this is a retrospective study, and although we included a relatively large number of cases for this rare disease, the chemotherapy regimens and radiation doses for patients were not uniform. Second, we did not find valid predictors of efficacy and did not perform an evaluation of side effects such as irradiation-induced vocal cord paralysis, mucositis, hypothyroidism, dermatitis, and pharyngitis. Therefore, it is better to be validated in further prospective studies.

## Conclusion

In conclusion, our study is a retrospective study of a larger sample of radical radiotherapy for cervical esophageal cancer. Our results show that the prognosis of cervical esophageal cancer is acceptable with concurrent radiotherapy and chemotherapy under IFI. Failure within the irradiated field was the predominant failure pattern. Therefore, the irradiation pattern of IFI is adequate and ENI is not necessary. Furthermore, the study showed that supraclavicular and upper mediastinum were the main sites of initial lymph node metastasis in cervical esophageal cancer. In contrast, the rate of lymph node metastasis was low in the upper cervical, 3P, and 6 regions and in the abdomen. The region of recurrent lymph nodes in cervical esophageal cancer is wide, including cervical, supraclavicular, upper mediastinal and abdominal lymph nodes.

## Data availability statement

The raw data supporting the conclusions of this article will be made available by the authors, without undue reservation.

## Ethics statement

The studies involving human participants were reviewed and approved by the Institutional Review Board of Fudan University Shanghai Cancer Center. The patients/participants provided their written informed consent to participate in this study.

## Author contributions

XZ, XF, PL, DL analyzed and interpreted the patient data. XZ and PL were major contributors in writing the manuscript. WZ, FS, HY and KZ put forward the research problems and carry out statistical analysis on the research problems. All authors read and approved the final manuscript.

## Funding

National Natural Science Foundation of China (grant number: 21172043), PI: KZ. National Natural Science Foundation of China (grant number: 21441010), PI: KZ. National Natural Science Foundation of China (grant number: 81872454), PI: KZ. Key Technologies Research and Development Program (grant number: 2016YFC1303200), PI: KZ.

## Conflict of interest

The authors declare that the research was conducted in the absence of any commercial or financial relationships that could be construed as a potential conflict of interest.

## Publisher’s note

All claims expressed in this article are solely those of the authors and do not necessarily represent those of their affiliated organizations, or those of the publisher, the editors and the reviewers. Any product that may be evaluated in this article, or claim that may be made by its manufacturer, is not guaranteed or endorsed by the publisher.

## Supplementary material

Supplementary Table 1 The number of chemotherapy cycles of 131 patients receiving intravenous chemotherapy The denominator is 131 patients receiving intravenous chemotherapy.

Supplementary Table 2 The pattern of treatment after recurrence. *The denominator is 90 who suffered recurrence.
