# Supplementary material for: Treatment outcomes of 156 patients with cervical esophageal cancers treated with definitive radiation therapy- A single-institution experience of a rare cancer
Source: Front Oncol. 2022 Aug 18;12:929583. doi: 10.3389/fonc.2022.929583 (PMC9435437; doi:10.3389/fonc.2022.929583)
Supplement: Supplementary Table 1 — The number of chemotherapy cycles of 131 patients receiving intravenous chemotherapy The denominator is 131 patients receiving intravenous chemotherapy. [file Table_1.pdf]

SupplementTable1：The number of chemotherapy cycles of 131 patients receiving intravenous chemotherapy

| | Patient Number | Percentage |
|---|---|---|
| 1 Cycle | 17 | 13.0 |
| 2 Cycles | 25 | 19.1 |
| 3 Cycles | 15 | 11.5 |
| More than 4 Cycles | 74 | 56.5 |

The denominator is 131 patients receiving intravenous chemotherapy

SupplementTable2：　The pattern of treatment after recurrence

| | Number | Proportion* |
|---|---|---|
| Chemotherapy | 16 | 17.8 |
| Radiotherapy | 6 | 6.7 |
| Surgery | 6 | 6.7 |
| Radiotherapy and chemotherapy | 12 | 13.3 |
| Radiotherapy and chemotherapy and Surgery | 1 | 1.1 |
| Palliative treatment | 49 | 54.4 |

*The denominator is 90 who suffered recurrence.
